# Unsuppressed lipolysis in adipocytes is linked with enhanced gluconeogenesis and altered bile acid physiology in *Insr*^*P1195L*/+^ mice fed high-fat-diet

**DOI:** 10.1038/srep17565

**Published:** 2015-11-30

**Authors:** Eun Young Lee, Kenichi Sakurai, Xilin Zhang, Chitoku Toda, Tomoaki Tanaka, Meizi Jiang, Takuji Shirasawa, Kaori Tachibana, Koutaro Yokote, Antonio Vidal-Puig, Yasuhiko Minokoshi, Takashi Miki

**Affiliations:** 1 Department of Medical Physiology, Chiba University, Graduate School of Medicine, Chiba 260-8670 Japan; 2 Department of Clinical Cell Biology and Medicine, Chiba University, Graduate School of Medicine, Chiba 260-8670 Japan; 3 Department of Developmental Physiology, National Institute for Physiological Sciences, Okazaki 444-8585, Japan; 4 Department of Genome Research and Clinical Application, Chiba University, Graduate School of Medicine, Chiba 260-8670 Japan; 5 Department of Ageing Control, Juntendo University, Graduate School of Medicine. Bunkyo 113-0033, Japan; 6 Department of Clinical Biochemistry, Metabolic Research Laboratories, Addenbrooke's Treatment Centre, Institute of Metabolic Science, University of Cambridge, Cambridge CB2 0QQ, UK

## Abstract

High-fat diet (HFD) triggers insulin resistance and diabetes mellitus, but their link remains unclear. Characterization of overt hyperglycemia in insulin receptor mutant (*Insr*^*P1195L/*+^) mice exposed to HFD (*Insr*^*P1195L/*+^/HFD mice) revealed increased glucose-6-phosphatase (*G6pc*) expression in liver and increased gluconeogenesis from glycerol. Lipolysis in white adipose tissues (WAT) and lipolysis-induced blood glucose rise were increased in *Insr*^*P1195L/*+^/HFD mice, while wild-type WAT transplantation ameliorated the hyperglycemia and the increased *G6pc* expression. We found that the expressions of genes involved in bile acid (BA) metabolism were altered in *Insr*^*P1195L/*+^/HFD liver. Among these, the expression of Cyp7a1, a BA synthesis enzyme, was insulin-dependent and was markedly decreased in *Insr*^*P1195L/*+^/HFD liver. Reduced *Cyp7a1* expression in *Insr*^*P1195L/*+^/HFD liver was rescued by WAT transplantation, and the expression of *Cyp7a1* was suppressed by glycerol administration in wild-type liver. These findings suggest that unsuppressed lipolysis in adipocytes elicited by HFD feeding is linked with enhanced gluconeogenesis from glycerol and with alterations in BA physiology in *Insr*^*P1195L/*+^/HFD liver.

The rapid increase in the prevalence of type 2 diabetes mellitus (T2DM) worldwide can be attributed to changes in environmental factors including less physical activity and over-nutrition. Excessive fat intake is known to promote onset of T2DM in genetically susceptible humans and rodents^1^. However, there are few animal models that exhibit normal glucose tolerance under normal diet but develop overt hyperglycemia in response to high-fat diet (HFD)^2^. Partly for this reason, the pathophysiology of HFD-induced diabetes mellitus has not been fully elucidated. T2DM is a metabolic disorder characterized by a chronic rise in blood glucose levels, principally due to insufficient insulin secretion and/or action. Insulin resistance is a pathogenic component of the disease, and can be induced both genetically and environmentally^3^. Genetic mutation or disruption of the insulin receptor (*Insr*) has been shown to cause insulin resistance and T2DM in both humans and mice^3^,^4^. In mice, whole body, homozygous deletion of the *Insr* gene results in neonatal lethality, which hinders studies on the pathophysiological relevance of the insulin receptor. As the insulin receptor is expressed in all cell types and insulin signaling mediates diverse cellular functions, the detailed insulin function of each tissue has been extensively studied using tissue-specific *Insr* knockout mice^3^,^5^, in which *Insr* expression is almost completely disrupted in a specific cell type.

A number of studies have shown that the various insulin signaling pathways affected by insulin resistance are not homogeneously affected^3^. Kahn *et al.* categorized such pathways into those remaining ‘insulin sensitive’ and those becoming ‘insulin resistant’, according to their relevance in Metabolic Syndrome. The concept of ‘selective insulin resistance’ is therefore critical for understanding the complex pathophysiology of T2DM, in which the insulin resistant state prevails in many tissues, but in a tissue-dependent, pathway-specific manner. Nevertheless, it has not been fully clarified which pathways contribute crucially to the development of T2DM.

In the present study, we examined changes in glucose metabolism in a mouse systemically harboring a loss of function mutation in *Insr* (a single amino acid substitution from proline to leucine at 1195 amino acid residue (P1195L)), which has been shown to act as a dominant-negative mutant in heterozygosity^6^. Heterozygous mutant (*Insr*^*P1195L/*+^) mice exhibit marked insulin resistance but avoid glucose intolerance even by glucose loading test^6^. Since this heterozygous mutation in *Insr* alone was insufficient to induce defective glucose homeostasis, we challenged the mice with HFD. Interestingly, *Insr*^*P1195L/*+^ mice were extremely susceptible to overt hyperglycemia by HFD. The mechanism of HFD-induced hyperglycemia was therefore investigated in *Insr*^*P1195L/*+^ mice.

Our findings reveal a concerted mechanism linking exacerbated lipolysis in WAT and increased gluconeogenesis from glycerol in liver of *Insr*^*P1195L/*+^/HFD mice, ultimately leading to the development of overt hyperglycemia. We also identified alterations in bile acid (BA) physiology in the liver of *Insr*^*P1195L/*+^/HFD mice.

## Results

### HFD feeding induces overt hyperglycemia and prevents body weight gain in *Insr* ^*P1195L/*+^ mice

**Figure 1:**
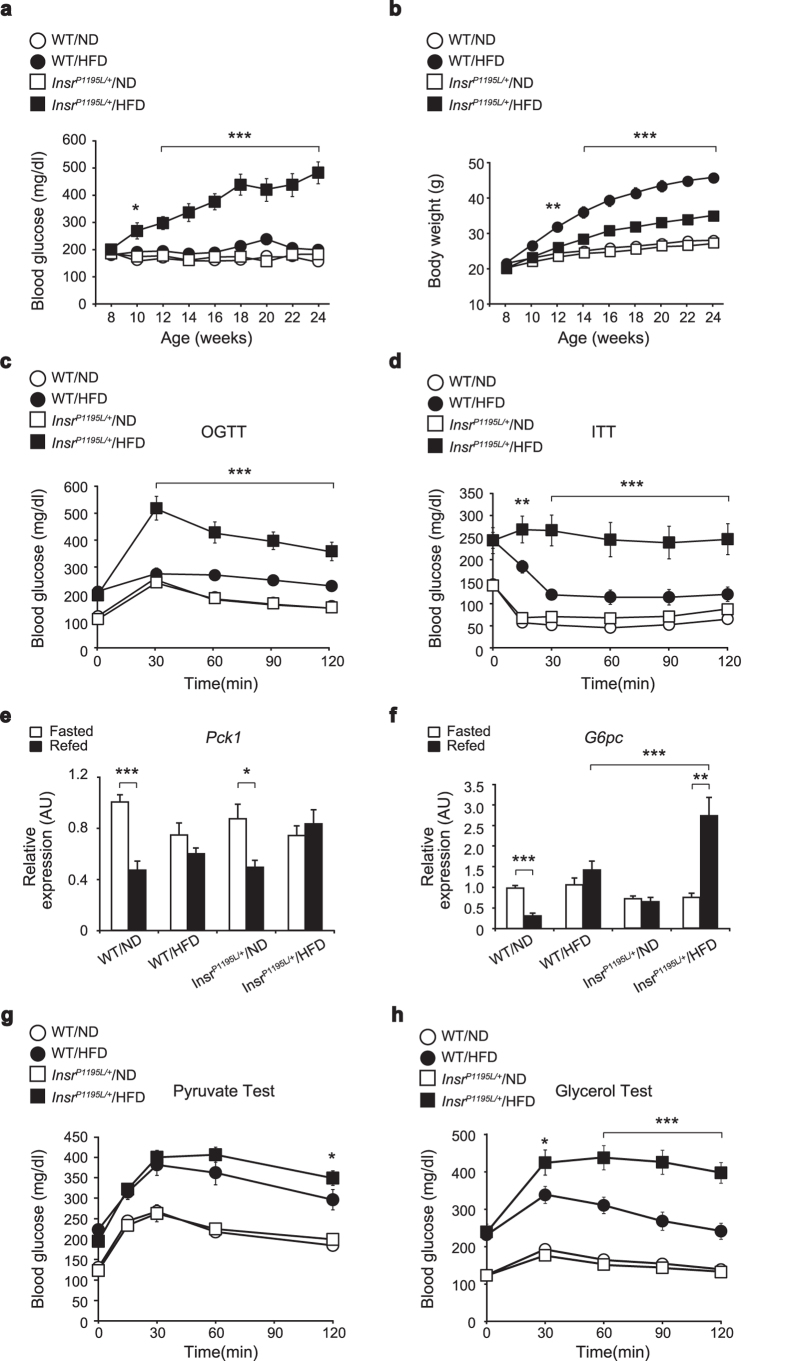
*Insr*^*P1195L*/+^/HFD mice exhibit glucose intolerance, insulin resistance, and increased gluconeogenesis from glycerol. (**a**) Blood glucose levels of *Insr*^*P1195L/*+^ and WT mice fed *ad libitum* (*n* = 8–10 per each group). (**b**) Body weight of *Insr*^*P1195L/*+^ and WT mice (*n* = 8–10 per each group). (**c**) OGTT (*n* = 8–10 per each group). (**d**) ITT (*n* = 6–10 per each group). (**e,f**) mRNA expressions of *Pck1* (**e**) and *G6pc* (**f**) in liver (*n* = 10–12 per each group). (**g,h**) Blood glucose levels after pyruvate (**g**) and glycerol (**h**) administration (*n* = 10–12 per each group). Data are mean ± SEM. Only the statistical difference between WT/HFD and *Insr*^*P1195L/*+^/HFD mice is depicted by asterisks in (**a–d,g,h**). Significance between strains (WT/HFD and *Insr*^*P1195L/*+^/HFD mice) at individual time points by two-tailed Student’s *t*-test (**a–d,g,h**). Two-way ANOVA plus Bonferroni *post-hoc* analysis (**e,f**). *P < 0.05, **P < 0.01, ***P < 0.001.

The blood glucose levels of *Insr*^*P1195L/*+^ mice under normal diet (ND) (*Insr*^*P1195L/*+^/ND mice) were not different from those of wild-type (WT) mice; however, *Insr*^*P1195L/*+^ mice under HFD (*Insr*^*P1195L/*+^/HFD mice) developed hyperglycemia (Fig. 1a). The expected increase in body weight of *Insr*^*P1195L/*+^/HFD mice was significantly blunted (Fig. 1b). Glucose intolerance in *Insr*^*P1195L/*+^/HFD mice was confirmed by oral glucose tolerance test (OGTT) (Fig. 1c). In addition, the glucose lowering effect of insulin was severely impaired in *Insr*^*P1195L/*+^/HFD mice, as assessed by insulin tolerance test (ITT) (Fig. 1d).

### Gluconeogenesis from glycerol but not from pyruvate is elevated in *Insr* ^*P1195L/*+^/HFD mice

Because severe insulin resistance in *Insr*^*P1195L/*+^/HFD mice suggested unsuppressed gluconeogenesis in an otherwise insulin resistant liver, as is found in liver-specific *Insr* knockout (LIRKO) mice^7^, mRNA expressions of two key enzymes, phosphoenolpyruvate carboxykinase 1 (*Pck1*) and glucose-6-phosphatase (*G6pc*), were examined (Fig. 1e,f). Although *Pck1* expression in *Insr*^*P1195L/*+^/HFD mice was not significantly different from that in WT/HFD mice, *G6pc* expression in *Insr*^*P1195L/*+^/HFD mice was markedly elevated by re-feeding. *Pck1* is a key enzyme involved in gluconeogenesis from amino acids and pyruvate, while *G6pc* participates in gluconeogenesis from glycerol as well as from amino acids and pyruvate. We therefore assessed gluconeogenesis from pyruvate (Fig. 1g) and glycerol (Fig. 1h) *in vivo* by measuring the blood glucose rise after intraperitoneal administration of either of the two substrates. Pyruvate administration increased the blood glucose levels similarly in *Insr*^*P1195L/*+^/HFD and WT/HFD mice (Fig. 1g). By contrast, glycerol administration to *Insr*^*P1195L/*+^/HFD mice evoked a larger increase in blood glucose levels compared with those of WT/HFD mice (Fig. 1h), suggesting that gluconeogenesis from glycerol but not from pyruvate was elevated in *Insr*^*P1195L/*+^*/*HFD mice.

### Fat accumulation is decreased in *Insr* ^*P1195L/*+^/HFD mice compared with that in WT/HFD mice

**Figure 2:**
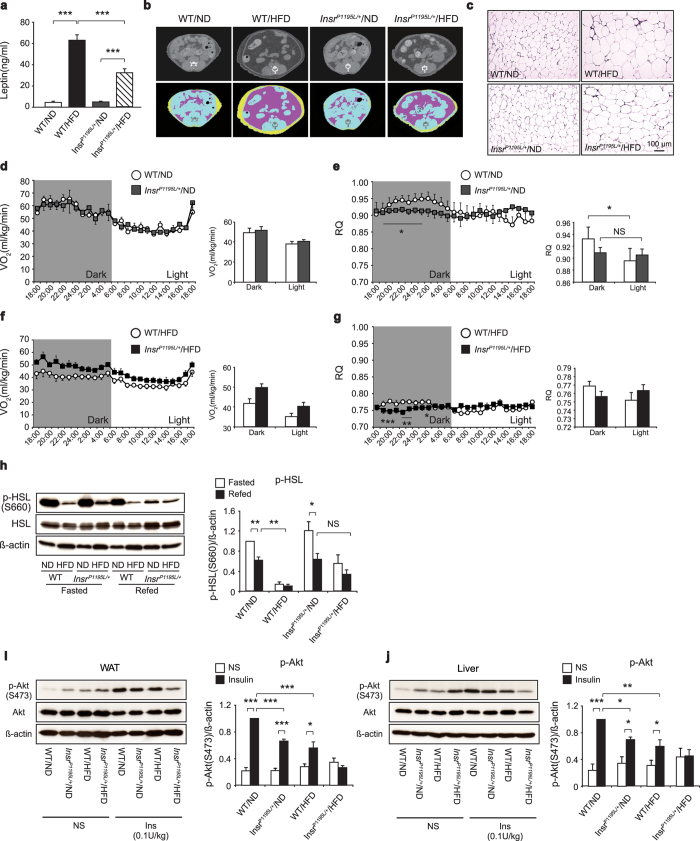
Fat combustion *in vivo* and lipolysis in WAT under HFD are increased in *Insr*^*P1195L/*+^ mice. (**a**) Serum leptin levels at 16 weeks (*n* = 8–10 per each group). (**b**) Representative CT images (22week of age) at the level of lower end of right kidney. Visceral and subcutaneous fat are indicated in pink and yellow, respectively. (**c**) Hematoxylin and eosin staining of epididymal fat. (**d–g**) Oxygen consumption rate (**d**) and RQ (**e**) under ND in *Insr*^*P1195L/*+^ and WT mice. Oxygen consumption rate (**f**) and RQ (**g**) under HFD in *Insr*^*P1195L/*+^ and WT mice (*n* = 6–7 per each group). (**h**) Western blot analysis of phospho-HSL in fasted and refed conditions. (left) A representative result showing increased phospho-HSL in *Insr*^*P1195L/*+^/HFD mice. (right) Quantified result of phospho-HSL levels. (*n* = 6–9 per each group). (**i**) Western blot analysis of phospho-Akt induced by insulin (0.1 IU/kg *i.v.*) in WAT. (left) A representative result showing attenuated phospho-Akt in *Insr*^*P1195L/*+^/HFD mice. (right) Quantified result of phospho-Akt levels. (*n* = 4–6 per each group). (**j**) Western blot analysis of phospho-Akt induced by insulin (0.1 IU/kg *i.v.*) in liver. (left) A representative result showing attenuated phospho-Akt in *Insr*^*P1195L/*+^/HFD mice. (right) Quantified result of phospho-Akt levels. (*n* = 4–6 per each group). Cropped blots were used. Full-length blots are presented in Supplementary Fig. S6. Data are mean ± SEM. Two-way ANOVA plus Bonferroni *post-hoc* analysis (**a**). Significance between treatment and strains by two-tailed Student’s *t*-test (**h–j**) and by One-way ANOVA plus Bonferroni *post-hoc* analysis (d-g, h-j), respectively. *P < 0.05, **P < 0.01, ***P < 0.001, NS; not significant.

Glycerol is produced by lipolysis of the triacylglycerols (TG) accumulated in the main energy reservoir, the white adipose tissues (WAT). When compared with WT/HFD mice, *Insr*^*P1195L/*+^/HFD mice had lower body weight (Fig. 1b), lower serum leptin levels (Fig. 2a), and less subcutaneous and visceral fat as assessed by computed tomography (CT) scanning (Fig. 2b), indicating that *Insr*^*P1195L/*+^/HFD mice have reduced fat mass. Histological analysis of epididymal fat revealed that *Insr*^*P1195L/*+^/HFD mice had smaller adipocytes than those of WT/HFD mice, while there was no difference in adipocyte size between *Insr*^*P1195L/*+^/ND and WT/ND mice (Fig. 2c, Supplementary Fig. S1a–d). In addition, the liver of *Insr*^*P1195L/*+^/HFD mice had lower TG content (Supplementary Fig. S2a) and less lipid accumulation in hepatocytes compared with WT/HFD mice (Supplementary Fig. S2c), indicating that ectopic fat accumulation is not a major cause of hyperglycemia in *Insr*^*P1195L/*+^/HFD mice. Alternatively, there was a positive correlation between body weight and TG content in all animal groups (Supplementary Fig. S2b).

### Respiratory quotient (RQ) is increased during dark phase in *Insr* ^*P1195L/*+^ mice under both ND and HFD

The decrease in fat accumulation in WAT and liver of *Insr*^*P1195L/*+^/HFD mice suggested that these animals may have increased energy dissipation and/or fat usage. We therefore measured the oxygen consumption rate and RQ in *Insr*^*P1195L/*+^ and WT mice (Fig. 2d–g). Although the oxygen consumption rate was not different between *Insr*^*P1195L/*+^ and WT mice under ND (Fig. 2d), *Insr*^*P1195L/*+^/ND mice exhibited lower RQ during dark phase compared with WT/ND mice (Fig. 2e). Although the oxygen consumption rate in *Insr*^*P1195L/*+^/HFD mice was plotted higher than that in WT/HFD mice when expressed per body weight (Fig. 2f), this difference is considered to be due to the significant decrease in their body weight. Nevertheless, the RQ during dark phase of *Insr*^*P1195L/*+^/HFD mice was lower than that of WT/HFD mice (Fig. 2g), suggesting the increased fat usage in these mice. These results also indicate that the diurnal metabolic switching of energy source by insulin is impaired in the state.

### Lipolysis is increased in *Insr* ^*P1195L/*+^ mice only under HFD

We then examined whether lipolysis was increased in *Insr*^*P1195L/*+^/HFD mice. Lipolysis in WAT is activated by β-adrenergic signaling and inhibited by insulin signaling via regulation of intracellular cAMP levels. Insulin suppresses lipolysis through PKA-dependent inactivation of hormone sensitive lipase (HSL)^8^. However, phospho-HSL levels in WAT of *Insr*^*P1195L/*+^/ND mice were increased after fasting and were decreased by re-feeding similarly to those of WT/ND mice (Fig. 2h). Importantly, HFD feeding markedly suppressed phospho-HSL levels in WT mice, but suppressed them much less in *Insr*^*P1195L/*+^ mice (Fig. 2h), indicating that the defective insulin signaling in WAT resulted in the increased HSL phosphorylation in *Insr*^*P1195L/*+^ mice preferentially under HFD condition.

### Akt phosphorylation by insulin is impaired in *Insr* ^*P1195L/*+^/HFD mice, both in WAT and in liver

Insulin inhibits lipolysis in adipocytes and gluconeogenesis in hepatocytes. Since lipolysis and gluconeogenesis were increased in *Insr*^*P1195L/*+^/HFD mice, we evaluated insulin signaling in WAT and liver by analyzing Akt phosphorylation in response to insulin administration *in vivo* (Fig. 2i,j). Quantification of phospho-Akt protein revealed that insulin-induced Akt phosphorylation was significantly less in *Insr*^*P1195L/*+^/ND mice and in WT/HFD mice compared with that in WT/ND mice. Notably, Akt phosphorylation in *Insr*^*P1195L/*+^/HFD mice was barely induced by insulin, indicating that insulin signaling is markedly impaired by combination of genetic mutation (*Insr*^*P1195L*^) in the insulin receptor and environmental condition (HFD feeding) in both WAT and liver.

### Lipolysis in the primary adipocytes of *Insr* ^*P1195L/*+^ mice is suppressed under ND but increased under HFD

**Figure 3:**
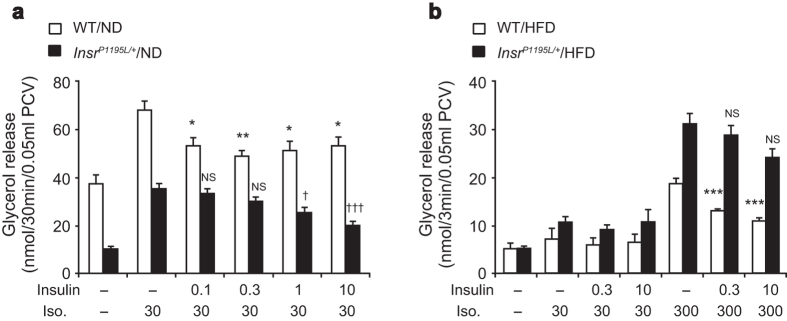
Lipolysis in primary adipocytes is increased in *Insr*^*P1195L/*+^/HFD mice. (**a,b**) Glycerol release of primary adipocytes isolated from *Insr*^*P1195L/*+^ and WT mice under ND (**a**) and HFD (**b**) (*n* = 6 per each group). Data are mean ± SEM. *, ^†^; comparison against isoproterenol-stimulated glycerol release (in the absence of insulin) in WT and *Insr*^*P1195L/*+^ mice, respectively. One-way ANOVA plus Bonferroni *post-hoc* analysis. Iso; isoproterenol, *P < 0.05, **P < 0.01, ***P < 0.001, ^†^*P *< 0.05, ^†††^*P *< 0.001, NS; not significant.

To exclude the influence of the sympathetic nervous system and hyperinsulinemia in *Insr*^*P1195L/*+^ mice, we measured glycerol release in primary adipocytes *in vitro*. Lipolysis was measured directly using isolated primary adipocytes (Fig. 3a,b). In *Insr*^*P1195L/*+^/ND mice, the basal and isoproterenol-stimulated lipolysis were significantly lower than that in WT/ND mice (Fig. 3a). By contrast, the inhibition of lipolysis by insulin was attenuated in *Insr*^*P1195L/*+^/ND mice. As a result, the lipolytic activity of *Insr*^*P1195L/*+^/ND mice in the presence of isoproterenol plus insulin was lower than that of WT/ND mice. HFD feeding blunted the sensitivity of isoproterenol-stimulated lipolysis in both WT and *Insr*^*P1195L/*+^ mice (Fig. 3b). Therefore, in mice under HFD, the anti-lipolytic action of insulin was evaluated in adipocytes stimulated with a higher dose (300 nM) of isoproterenol. Although the basal lipolysis in *Insr*^*P1195L/*+^/HFD adipocytes was similar to that in WT/HFD adipocytes, isoproterenol-stimulated lipolysis was significantly higher in *Insr*^*P1195L/*+^/HFD adipocytes, and insulin suppressed glycerol release poorly (Fig. 3b). The increased lipolysis by isoproterenol alone (in the absence of co-treatment with insulin) in *Insr*^*P1195L/*+^/HFD adipocytes also suggests that some factors [such as activities of perilipin^9^ and/or adipocyte triglyceride lipase^10^ (ATGL)] other than insulin-dependent suppression of phospho-HSL may contribute to the increased lipolysis^11^.

### Stimulation of lipolysis by CL316432 evokes hyperglycemia in *Insr* ^*P1195L/*+^/HFD mice

**Figure 4:**
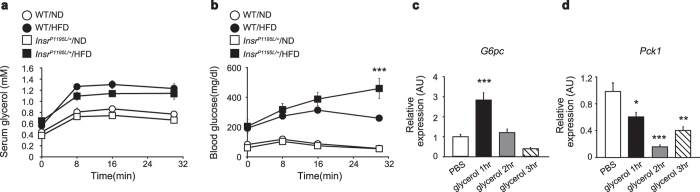
Lipolysis-induced gluconeogenesis is increased in *Insr*^*P1195L/*+^/HFD mice. (**a,b**) Serum glycerol (**a**) and blood glucose levels (**b**) after CL316432 administration (*n* = 5–10 per each group). (**c,d**) Expression of *G6pc* (**c**) and *Pck1* (**d**) in the WT liver after intraperitoneal glycerol administration (*n* = 7–10 per each group). Data are mean ± SEM. Only the statistical difference between WT/HFD and *Insr*^*P1195L/*+^/HFD mice is depicted by asterisk in b. Significance between strains (WT/HFD and *Insr*^*P1195L/*+^/HFD mice) at individual time points by two-tailed Student’s *t*-test (**a,b**) One-way ANOVA plus Bonferroni *post-hoc* analysis (**c,d**). *P < 0.05, **P < 0.01, ***P < 0.001.

We also examined whether the increase in lipolysis could contribute to hyperglycemia in *Insr*^*P1195L/*+^/HFD mice under *in vivo* conditions. We treated the mice with CL316432, a β3-adrenergic receptor-specific agonist, and monitored the changes in serum glycerol (Fig. 4a) and blood glucose (Fig. 4b) levels. Although the serum glycerol levels were similarly increased in WT/HFD mice and *Insr*^*P1195L/*+^/HFD mice, the blood glucose levels after CL316432 administration were significantly higher in *Insr*^*P1195L/*+^/HFD mice, indicating that enhanced lipolysis exacerbates hyperglycemia in *Insr*^*P1195L/*+^/HFD mice.

Our results also indicate increased gluconeogenesis from glycerol. *G6pc* is a key enzyme that mediates gluconeogenesis from glycerol and its gene expression is suppressed by insulin. Increased *G6pc* expression in *Insr*^*P1195L/*+^/HFD liver could therefore result from impaired repression by insulin in liver and/or be secondary to increased glycerol influx to liver. Therefore, we examined whether glycerol administration increases *G6pc* expression in WT liver *in vivo* (Fig. 4c). Glycerol significantly increased the expression of *G6pc* while *Pck1* expression was decreased by glycerol administration (Fig. 4d), suggesting that the intracellular abundance of substrates for gluconeogenesis determines the expressions of their regulatory enzymes.

### Transplantation of wild-type subcutaneous WAT ameliorates hyperglycemia of *Insr* ^*P1195L/*+^/HFD mice

**Figure 5:**
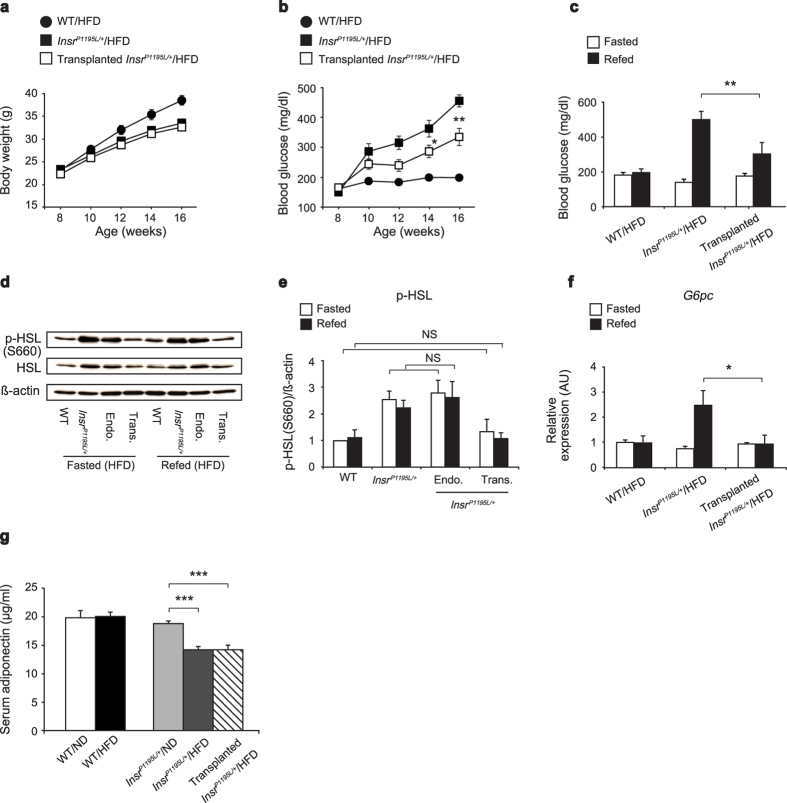
Hyperglycemia in *Insr*^*P1195L/*+^/HFD mice is ameliorated by transplantation of wild-type subcutaneous WAT. (**a,b**) Body weight (**a**) and blood glucose levels (**b**) (*n* = 13–15 per each group). (**c**) The blood glucose levels at 16-hr fasted and 3 hrs after food replenishment at 18–20 weeks of age (*n* = 7–10 per each group). (**d,e**) Western blot analysis of phospho-HSL levels in fasted and refed conditions. (**d**) A representative result showing increased phospho-HSL in *Insr*^*P1195L/*+^/HFD mice. Endo., endogenous fat; Trans., transplanted fat. Cropped blots were used. Full-length blots are presented in Supplementary Fig. S6. (**e**) Quantified result of phospho-HSL levels. (*n* = 4 per each group). (**f**) mRNA expressions of *G6pc* in liver on fasted and refed conditions (*n*v6–8 per each group). (**g**) Serum adiponectin levels (*n* = 8 per each group). Data are mean ± SEM. Significance between treatment (un-transplanted *Insr*^*P1195L/*+^/HFD and transplanted *Insr*^*P1195L/*+^/HFD mice) at individual time points by two-tailed Student’s *t*-test (**a–c,f**). One-way ANOVA plus Bonferroni *post-hoc* analysis (**e,g**). *P < 0.05, **P < 0.01, ***P < 0.001, NS; not significant.

To assess the involvement of increased lipolysis in WAT of *Insr*^*P1195L/*+^/HFD mice on the development of hyperglycemia, we transplanted wild-type subcutaneous WAT to *Insr*^*P1195L/*+^ mice. Although transplanted *Insr*^*P1195L/*+^/HFD mice gained weight similarly to un-transplanted *Insr*^*P1195L/*+^/HFD mice (Fig. 5a), the blood glucose levels in fed conditions were significantly lower than those of un-transplanted *Insr*^*P1195L/*+^/HFD mice at 14 and 16 weeks of age (Fig. 5b). In addition, the rise in blood glucose levels of transplanted *Insr*^*P1195L/*+^/HFD mice on re-feeding was significantly reduced compared with those of un-transplanted *Insr*^*P1195L/*+^/HFD mice (Fig. 5c). As the phospho-HSL level in the transplanted fat pad was similar to that of endogenous WAT in WT/HFD mice (Fig. 5d,e), suppression of lipolysis in the transplant might well have ameliorated the systemic hyperglycemia of *Insr*^*P1195L/*+^/HFD mice. As expected, the increased *G6pc* expression in *Insr*^*P1195L/*+^/HFD liver on re-feeding was markedly reduced by the transplantation of wild-type subcutaneous fat to the mice (Fig. 5f). To assess the involvement of anti-inflammatory cytokines released from the transplant, we measured serum adiponectin levels (Fig. 5g). In our experimental conditions with 45% HFD, adiponectin was not decreased in WT/HFD mice. By contrast, adiponectin of *Insr*^*P1195L/*+^/HFD mice was significantly lower than that of *Insr*^*P1195L/*+^/ND mice. Notably, fat transplantation to *Insr*^*P1195L/*+^/HFD mice did not increase adiponectin, suggesting that change in adiponectin did not contribute to improved glycemia.

### BA physiology is diversely altered in *Insr* ^*P1195L/*+^/HFD mice

**Figure 6:**
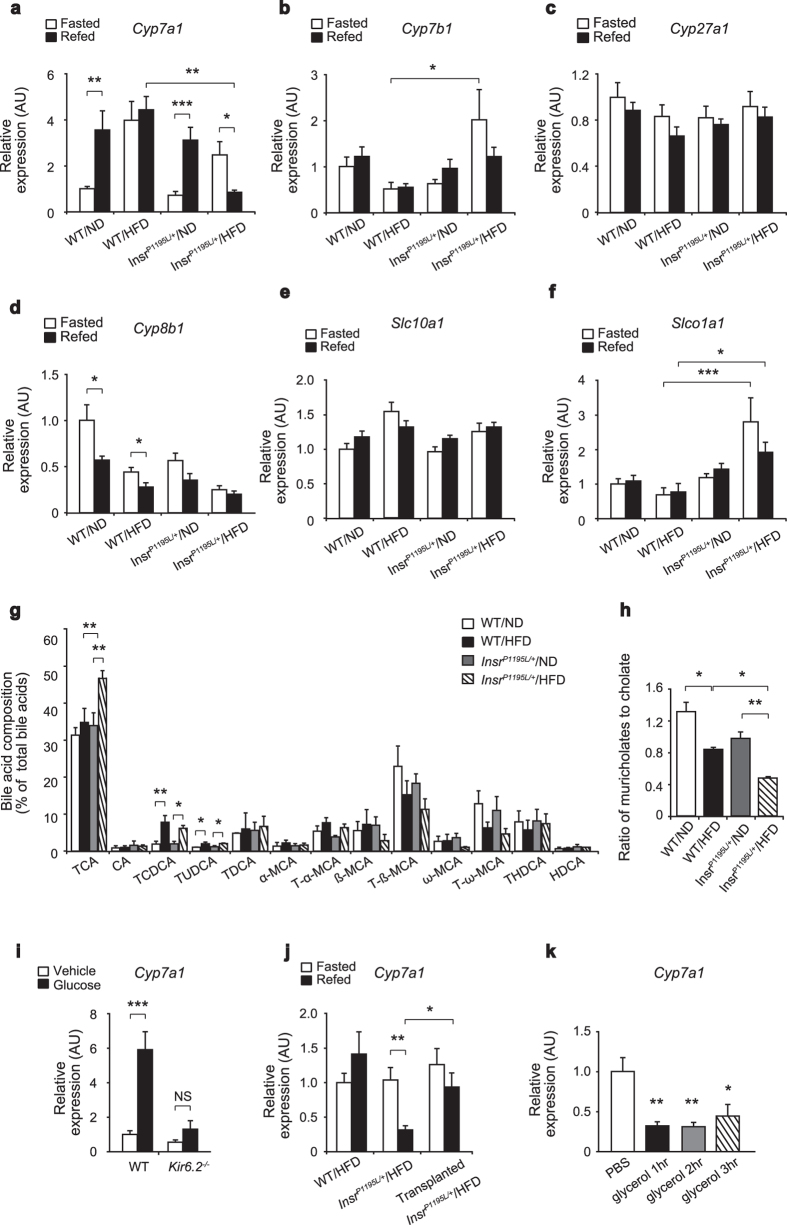
BA physiology is diversely altered in *Insr*^*P1195L/*+^/HFD liver. (**a–d**) mRNA expressions of genes involved in BA synthesis (*n* = 8–12 per each group). (**e,f**) mRNA expressions of genes involved in BA transport (*n* = 8–12 per each group). (**g**) Relative BA composition in liver in refed condition (*n* = 3). (**h**) The ratio of muricholates to cholates, calculated with the molar percentage of tauro α-, tauro β-, and tauro ω-mucricholates and taurocholate. (**i**) mRNA expression of *Cyp7a1* after oral glucose loading in *Kir6.2*^−/−^ mice (*n* = 7–12 per each group). (**j**) mRNA expression of *Cyp7a1* in fat transplanted *Insr*^*P1195L/*+^/HFD mice (*n* = 5–7 per each group). (**k**) mRNA expression of *Cyp7a1* in the WT liver after intraperitoneal glycerol administration (*n* = 7–10 per each group). Data are mean ± SEM. Two-way ANOVA plus Bonferroni *post-hoc* analysis (**a–f**). Significance between treatment by two-tailed Student’s *t*-test (**i**). One-way ANOVA plus Bonferroni *post-hoc* analysis **(g,h,j,k**). *P < 0.05, **P < 0.01, ***P < 0.001, NS; not significant.

As fat-specific insulin receptor deficient mice (FIRKO mice) failed to exhibit glucose intolerance, we examined the contribution of insulin resistance in liver. To clarify its molecular mechanism, we performed microarray analysis in liver of *Insr*^*P1195L/*+^/HFD and WT/HFD mice, and found that expressions of several genes involved in BA physiology were altered. We therefore quantified mRNA expressions of the enzymes involved in BA synthesis (Fig. 6a–d) and BA transporters (Fig. 6e,f). Expression of Cyp7a1, the rate-limiting enzyme of BA synthesis, was significantly increased by re-feeding similarly in WT/ND mice and *Insr*^*P1195L/*+^/ND mice, while that in WT/HFD mice was significantly elevated under fasted condition, and did not show further increase by re-feeding. Interestingly, *Cyp7a1* expression in *Insr*^*P1195L/*+^/HFD mice was markedly decreased by re-feeding (Fig. 6a). By contrast, expression of Cyp7b1, another enzyme of BA synthesis, was increased in *Insr*^*P1195L/*+^/HFD mice (Fig. 6b). Expression of *Cyp27a1* was not altered among the 4 animal groups (Fig. 6c). Expression of Cyp8b1, the sterol 12α-hydroxylase required for generation of CA, was decreased in *Insr*^*P1195L/*+^/HFD mice (Fig. 6d). In addition, we examined expressions of BA transporters, Slc10a1 and Slco1a1 (Fig. 6e,f). Expression of *Slco1a1* but not *Slc10a1* was significantly increased in *Insr*^*P1195L/*+^/HFD mice.

We therefore measured the BA content in liver by LC-MS/MS analysis and found that BA composition was altered in *Insr*^*P1195L/*+^/HFD liver (Fig. 6g,h) as well as in serum (Supplementary Fig. S5a,b). In contrast, total BA content in *Insr*^*P1195L/*+^/HFD liver was not different from that in WT/HFD liver, suggesting that BA content might be maintained through various compensatory mechanisms, such as altered BA synthesis, absorption, and secretion. It remains undetermined whether or not the decreased *Cyp7a1* expression in *Insr*^*P1195L/*+^/HFD liver is the primary or a secondary change induced by HFD feeding plus insulin resistance. However, our present findings show that HFD feeding plus insulin resistance induces alterations in BA physiology in liver.

We then examined acute regulation of *Cyp7a1* expression in liver. We found that *Cyp7a1* expression was significantly increased by refeeding in WT/ND and *Insr*^*P1195L/*+^/ND mice. Both glucose and insulin signaling have been reported to regulate *Cyp7a1* expression in liver^12^. To clarify their relative importance, we examined *Cyp7a1* induction in response to oral glucose loading in *Kir6.2* deficient mice (*Kir6.2*^−/−^ mice), which lack glucose-stimulated insulin secretion^13^. Glucose loading failed to induce *Cyp7a1* expression in *Kir6.2*^−/−^ mice (Fig. 6i), suggesting that insulin plays a critical role in *Cyp7a1* expression. In addition, the effect on BA physiology of fat transplantation to *Insr*^*P1195L/*+^/HFD mice was assessed by measuring *Cyp7a1* in transplanted *Insr*^*P1195L/*+^/HFD mice (Fig. 6j). Reduced mRNA expression of *Cyp7a1* in *Insr*^*P1195L/*+^/HFD mice on re-feeding was significantly increased by fat transplantation, suggesting that modulation of metabolism in adipocytes by WAT transplantation may elicit alteration in BA physiology in liver. To test this, we examined whether *in vivo* administration of glycerol suppresses *Cyp7a1* in wild-type liver. Interestingly, intraperitoneal glycerol administration significantly inhibited *Cyp7a1* expression in liver (Fig. 6k), suggesting that the unsuppressed lipolysis in WAT might alter BA physiology in liver via glycerol dynamics.

**Figure 7:**
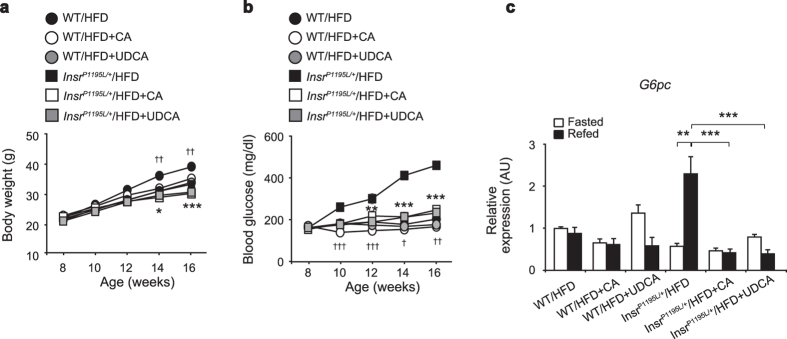
Supplementation with either CA or UDCA ameliorated hyperglycemia of *Insr*^*P1195L/*+^/HFD mice. (**a,b**) Changes in body weight (**a**) and blood glucose levels (**b**). (**c**) mRNA expressions of *G6pc* in liver in fasted and refed conditions (*n* = 5–8 per each group). ^*,†^only the statistical difference between supplementation with or without CA in *Insr*^*P1195L/*+^/HFD mice (*) and in WT/HFD (^†^) is depicted by symbols in (**a**,**b**). Data are mean ± SEM. Significance between treatment at individual time points by two-tailed Student’s *t*-test (**a,b**). Two-way ANOVA plus Bonferroni *post-hoc* analysis (**c**). ^*,†^*P *< 0.05, ^**,††^*P *< 0.01, ^***,†††^*P *< 0.001.

Recent studies have shown that BAs play an important role in the regulation of energy and glucose metabolism. Various molecules including the farnesoid X receptor (FXR)^14^ and the G-protein coupled receptor TGR5^15^ are known to be involved in the BA-medicated metabolic regulation. In accord with the previous reports^16^, we found that supplementation with CA significantly decreased the gain in body weight in both WT/HFD and *Insr*^*P1195L/*+^/HFD mice (Fig. 7a). In addition, supplementation with ursodeoxycholic acid (UDCA) elicited a reduced body weight gain similar to that by CA supplementation. Notably, the supplementation with either CA or UDCA restored euglycemia in *Insr*^*P1195L/*+^/HFD mice (Fig. 7b) and prevented the rise in *G6pc* expression on re-feeding (Fig. 7c), suggesting that alteration in BAs may influence glucose homeostasis in *Insr*^*P1195L/*+^/HFD mice.

## Discussion

We previously reported that mutant mice harboring a single amino acid substitution in the insulin receptor (*Insr*^*P1195L/*+^ mice) exhibit insulin resistance but normal glucose tolerance^6^. In the present study, we found that *Insr*^*P1195L/*+^ mice develop overt hyperglycemia and only mild obesity under HFD. In some ways, the lesser degree of obesity and exacerbated metabolic disturbances in these mice resembles the clinical characteristics of Asian patients with T2DM^17^,^18^, suggesting that *Insr*^*P1195L/*+^/HFD mice are suitable for investigating the pathophysiology of HFD-sensitive T2DM in humans.

The glucose intolerance and impaired glucose lowering effect of insulin in *Insr*^*P1195L/*+^/HFD mice resemble those in LIRKO mice^3^,^7^. Hyperglycemia in LIRKO mice is characterized by unsuppressed hepatic gluconeogenesis associated with increased mRNA expressions of two gluconeogenesis enzymes, *Pck1* and *G6pc*. Although *Pck1* expression was only mildly elevated, *G6pc* expression was markedly elevated in *Insr*^*P1195L/*+^/HFD mice. *Insr*^*P1195L/*+^/HFD mice exhibited enhanced gluconeogenesis from glycerol (Fig. 1h) but not from pyruvate (Fig. 1g), compared with WT/HFD mice. In addition, lipolysis and subsequent gluconeogenesis from glycerol was found to be increased in *Insr*^*P1195L/*+^*/*HFD mice. By contrast, the expression of fatty acid synthase (*Fas*) in liver, for example, was not decreased in *Insr*^*P1195L/*+^/HFD mice (Supplementary Fig. S3), although its transcription is known to be stimulated by insulin, principally through SREBP-1c^19^. In addition, *Srebp-1* expression in the liver by re-feeding was not attenuated in *Insr*^*P1195L/*+^/HFD mice, indicating that hepatic *Srebp-1* expression in the liver by re-feeding and by HFD feeding remains intact under insulin resistance (i.e., is insulin sensitive^3^). On the other hand, RQ during dark phase was significantly reduced in *Insr*^*P1195L/*+^ mice even under ND condition (Fig. 2e,g), suggesting that diurnal switching of the energy usage was inflexible under insulin resistance (i.e., is insulin resistant^3^). This metabolic inflexibility in *Insr*^*P1195L/*+^ mice could be also due to compensatory increase in fat utilization in fat and skeletal muscle during dark phase caused by impaired glucose uptake in these tissues.

We found that fat transplantation significantly ameliorated hyperglycemia in *Insr*^*P1195L/*+^/HFD mice. We also found that serum FFA levels in *Insr*^*P1195L/*+^/HFD mice were significantly higher than those in WT/HFD mice (Supplementary Fig. S4), suggesting that pro-inflammatory factors may participate in hyperglycemia of *Insr*^*P1195L/*+^/HFD mice in addition to the unsuppressed glycerol release.

With regard to the amelioration of hyperglycemia in fat-transplanted *Insr*^*P1195L/*+^/HFD mice, it is unclear whether the improved glucose uptake into the transplant may play a major role, as WAT has been reported to play a minor role in glucose uptake in rodents^20^. However, increased glucose uptake and elevated lipogenesis in WAT has been found to improve glucose tolerance through synthesis of branched fatty acid esters of hydroxy fatty acids (FAHFAs)^21^. Although the presence of the insulin-sensitive fat transplant might increase the FAHFAs in fat-transplanted *Insr*^*P1195L/*+^/HFD mice, our present results suggest that normal glycerol metabolism in WAT is also important for the maintenance of glucose homeostasis under insulin resistance. Nevertheless, the lack of glucose intolerance in FIRKO mice suggested that insulin resistance in tissues other than WAT is involved in developing hyperglycemia in *Insr*^*P1195L/*+^/HFD mice. Importantly, liver-specific insulin receptor-deficient mice (LIRKO mice) exhibit hyperglycemia associated with increased *G6pc* expression^7^. Although insufficient insulin secretion also is likely to contribute to the hyperglycemia in *Insr*^*P1195L/*+^/HFD mice, as found in both humans and rodents^22^, in the present study, we focused on the involvement of insulin resistance in liver. Notably, we found that the expressions of genes involved in BA metabolism are diversely altered. Quantification of BA composition revealed that BAs of *Insr*^*P1195L/*+^/HFD liver were more hydrophobic than those of WT/HFD liver. However, it remains unknown whether BA hydrophobicity is related to the hyperglycemia in *Insr*^*P1195L/*+^/HFD mice. It has been reported that hydrophobicity in liver is increased in LIRKO mice, which develop gallstones under lithogenic diet^23^. In contrast, our present study suggests that HFD feeding plus hepatic insulin resistance is involved in the alterations in BA composition.

Hepatic expression of *Cyp7a1* was dramatically reduced in *Insr*^*P1195L/*+^/HFD mice. Interestingly, in LIRKO mice, *Cyp7a1* expression was also markedly decreased, suggesting that insulin signaling is involved in *Cyp7a1* expression^23^. Although *Cyp7a1* expression is reported to be induced acutely by glucose and insulin^12^, our study in *Kir6.2*^−/−^ mice, which lack glucose-stimulated insulin secretion, revealed the importance of insulin in its transactivation. In addition, Cyp7a1-overexpressing transgenic mice have been reported to show improved glucose tolerance and reduced *G6pc* expression under HFD condition^24^. Furthermore, tauro-CA (TCA) has been reported to suppress *G6pc* expression in primary hepatocytes^25^. Reduction of blood glucose levels and suppression of *G6pc* expression in liver of *Insr*^*P1195L/*+^/HFD mice by supplementation with either CA or UDCA suggest that alterations in BAs might contribute to the development of hyperglycemia in these mice. Amelioration of *Cyp7a1* and *G6pc* by fat transplantation in *Insr*^*P1195L/*+^/HFD liver and suppression of *Cyp7a1* by glycerol in wild-type mice suggest that suppressed hepatic expression of *Cyp7a1* might well play a role in the regulation of glucose homeostasis in *Insr*^*P1195L/*+^/HFD mice. Although the underlying mechanisms of BA physiology in glucose metabolism remain unknown, many studies have found that BA physiology is altered in T2DM patients^26^. Considering that FXR signaling is activated by CA but not by UDCA, the anti-diabetic action of BAs in *Insr*^*P1195L/*+^/HFD mice might be mediated also by a FXR-independent mechanism.

**Figure 8:**
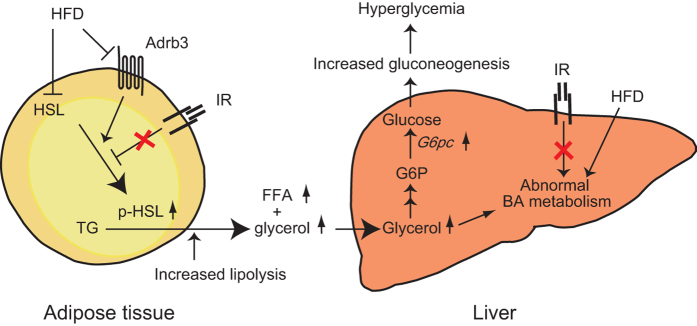
Model for pathophysiological mechanism of hyperglycemia in *Insr*^*P1195L/*+^*/*HFD mice. See text for detail.

In conclusion, excessive glycerol supply from WAT is suggested to induce enhanced *G6pc* expression, leading to unsuppressed gluconeogenesis and resulting in hyperglycemia in *Insr*^*P1195L/*+^/HFD mice. In addition, alterations of metabolism in adipocytes are linked with abnormal BA physiology in *Insr*^*P1195L/*+^/HFD mice (Fig. 8).

## Methods

### Animals and metabolic analyses

*Insr*^*P1195L/*+^ mice were generated as described previously^27^. The mice have been backcrossed to the C57BL/6 strain over more than 10 generations. Mice were fed *ad libitum* and kept on a 12-hr light/12-hr dark cycle. For ND feeding, the mice were maintained on standard chow (CE-2) (12.1 % kcal from fat) (Clea Japan Inc., Tokyo, Japan). For HFD feeding, the mice were maintained on a HFD (D12492) (60.0 % kcal from fat) (Research Diets Inc., New Brunswick, NJ, USA) starting at 8 weeks of age unless stated otherwise. OGTT and ITT were performed as previously described^13^, using 1 g/kg glucose and 0.75 IU/kg insulin after 16-hr fast. For pyruvate tolerance test, glycerol tolerance test, and CL316432 loading test, 16-hr fasted mice were administered intraperitoneally with 1 g/kg sodium pyruvate, 0.5 g/kg glycerol, and 0.1 mg/kg CL316432 (Tocris Bioscience, Bristol, UK), respectively, and blood glucose levels were monitored at the indicated time points. Blood glucose was measured as previously described^13^. Glycerol in the serum was measured using the kit from BioVision, Inc. (San Francisco, CA, USA). Serum adiponectin levels were measured using the kit from Otsuka pharmaceuticals. (Tokyo, Japan). For CA and UDCA supplementation, CA (0.25 % *wt/wt*) (Sigma, St. Louis, MO, USA) or UDCA (0.25 % *wt/wt*) (Tokyo chemical industry, Tokyo, Japan) was added to the HFD (D12492), and the diet was given to the mice at 8 weeks of age for 8 weeks. All animal studies were conducted in accordance with the International Guiding Principles for Biomedical Research Involving Animals and were approved by the Animal Care and Use Committees of Chiba University and the National Institute for Physiological Sciences in Japan.

### Real-time qRT-PCR analyses

Total RNA was isolated from WAT (epididymal fat pad, unless stated otherwise) and liver and subjected to qRT-PCR analyses using SYBR Green, according to the manufacturer’s protocol. The primers used were 5′-caccatcacctcctggaaga-3′ and 5′-gggtgcagaatctcgagttg-3′ for *Pck1*, 5′-gtggcagtggtcggagact-3′ and 5′-acgggcgttgtccaaac-3′ for *G6pc*, 5′-tacagagtgctggccaagag-3′ and 5′-ttcaaggatgcactggagag-3′ for *Cyp7a1*, 5′-tagccctctttcctccactcata-3′ and 5′-gaagcgatcgaacctaaattcct-3′ for *Cyp7b1*, 5′-gcctcacctatgggatcttca-3v and 5v-tcaaagcctgacgcagatg-3v for *Cyp27a1*, 5′-ggctggcttcctgagcttatt-3′ and 5′-acttcctgaacagctcatcgg-3′ for *Cyp8b1*, 5′-acctgtctaacctcttcacc-3′ and 5′-caatgctgaggttcatgtcc-3′ for *Slc10a1*, 5′-ggaagattgttggcccgatt-3′ and 5′-agtgggagttatggtcaggt-3v for *Slco1a1*, 5′-gcgtcgtgattagcgatga-3′ and 5′-atggcctcccatctcctt-3′ for *Hprt*.

### Western blotting

Western blotting analyses were carried out with tissue homogenates from WAT under standardized methods. To elucidate HSL phosphorylation of WAT in fasted and refed conditions, the mice were fasted for 16 hrs in fasted groups and refed for 3 hrs in refed groups. To elucidate insulin signaling in response to intravenous insulin, the cervical vein was exposed and 0.1 IU/kg insulin was injected via the vein. Five minutes later, WAT and liver were removed. The tissues were lysed in sonication buffer (20 mM HEPES pH7.5; 150 mM NaCl; 25 mM EDTA 1% NP-40; 10% glycerol, 1 mM sodium vanadate, 1 mM phenylmethylsulfonyl fluoride [PMSF] and protease and phosphatase inhibitors). Twenty μg protein for WAT and 50 μg protein for liver was subjected to SDS-PAGE. Antibodies against the following proteins were used (all from Cell Signaling): phospho-HSL (p-S660) (1:1000), HSL (1:1000), phospho-Akt (p-S473) (1:1000), Akt (1:1000) and β-actin (1:1000).

### Histological examination

Histological examination of WAT and liver was carried out under standardized methods. The tissues were dissected, fixed in 10% buffered formalin overnight, washed with PBS, and embedded in paraffin. Sections were stained with hematoxylin and eosin for histological analysis, and the slides were examined under a Keyence BZ-8100 microscope (Keyence Japan, Osaka, Japan).

### CT scanning

Mice were anesthetized and placed in the chamber of a CT scanner for mouse (Latheta LCT-200, Aloka, Tokyo, Japan). The CT scanner was calibrated according to the manufacturer’s protocols.

### Oxygen consumption

Oxygen consumption was measured using a MK-5000RQ (MK-5000RQ, Muromachi Kikai, Tokyo, Japan), with one mouse per chamber. Four groups of mice (*Insr*^*P1195L/*+^ and WT mice under either ND or HFD) aged 14 weeks (after 6 weeks of HFD feeding) were tested simultaneously. Mice had free access to food and water. Oxygen consumption was measured after the acclimatizing period for more than 2 weeks.

### Fat transplantation

Subcutaneous fat pad isolated from abdominal walls of 8-week-old male WT mice was transplanted into the subcutaneous area below the skin on the back of *Insr*^*P1195L/*+^ mice at 7 weeks of age, as previously reported^28^. The fat pad isolated from two donor mice (~0.7g of fat tissue/2 mice) was used for a single recipient. After one week of recovery period, only the recipient mice that exceeded the initial body weight (i.e., body weight of the recipient plus weight of the transplant) were subjected to a HFD (D12451) (45.0 % kcal from fat) (Research Diets Inc.) for 8 weeks. Sham operation was performed on the un-transplanted mice in the fat transplantation experiments (Fig. 5a–g).

### Isolation of mouse primary adipocytes and lipolysis assay

Epididymal fat was excised, minced, and digested with 0.2 % (*w/v*) collagenase type I (Life Technologies) for 30 min at 37 °C under shaking, as previously described^29^. The cells were passed through 500-μm nylon mesh and washed by Krebs Ringer Bicarbonate buffer 4 times. After 30 min pre-incubation, adipocytes were incubated with or without insulin (0.1 ~ 3 nM) for 5 min, and the cells were further incubated with isoproterenol (30 or 300 nM) for 30 min. The reaction solution was subjected to glycerol assay to assess the lipolysis activity. Glycerol in the buffer was measured using the kits from BioVision, Inc.

### Measurement of BA composition

Liver samples and serum for measuring BA composition were collected from the mice at 3 hrs after refeeding. BA composition was measured in Junshin Clinic Bile Acid Institute (Tokyo, Japan) by LC-MS/MS in reference to the method described previously^30^,^31^. The standard for tauro-αMCA-3-sulfate was kindly provided by Dr. Takashi Iida at Nippon University.

### Statistics

Results are expressed as means ± SEM. Differences between two groups were assessed using the unpaired two-tailed Student’s *t*-test. Data sets involving more than three groups were assessed by One-way ANOVA or Two-way ANOVA with Bonferroni *post-hoc* test. *P *< 0.05 was considered to be statistically significant.

## Additional Information

**How to cite this article**: Young Lee, E. *et al.* Unsuppressed lipolysis in adipocytes is linked with enhanced gluconeogenesis and altered bile acid physiology in *Insr*^*P1195L*/+^ mice fed high-fat-diet. *Sci. Rep.* **5**, 17565; doi: 10.1038/srep17565 (2015).

## Supplementary Material

Supplementary Information

## References

[b1] Nolan C. J., Damm P. & Prentki M. Type 2 diabetes across generations: from pathophysiology to prevention and management. The Lancet 378, 169–181 (2011). 10.1016/S0140-6736(11)60614-4 21705072

[b2] Nakamura A. *et al.* Protection from non-alcoholic steatohepatitis and liver tumourigenesis in high fat-fed insulin receptor substrate-1-knockout mice despite insulin resistance. Diabetologia 55, 3382–3391 (2012). 22955994 10.1007/s00125-012-2703-1

[b3] Biddinger S. B. & Kahn C. R. From mice to men: insights into the insulin resistance syndromes. Annu. Rev. Physiol. 68, 123–158 (2006). 16460269 10.1146/annurev.physiol.68.040104.124723

[b4] Semple R. K., Savage D. B., Cochran E. K., Gorden P. & O'Rahilly S. Genetic syndromes of severe insulin resistance. Endocr. Rev. 32, 498–514 (2011). 21536711 10.1210/er.2010-0020

[b5] Kadowaki T., Ueki K., Yamauchi T. & Kubota N. SnapShot: Insulin signaling pathways. Cell 148, 624 624.e1 (2012). 22304926 10.1016/j.cell.2012.01.034

[b6] Ogino J. *et al.* Insulin resistance and increased pancreatic beta-cell proliferation in mice expressing a mutant insulin receptor (P1195L). J. Endocrinol. 190, 739–747 (2006). 17003275 10.1677/joe.1.06849

[b7] Michael M. D. *et al.* Loss of insulin signaling in hepatocytes leads to severe insulin resistance and progressive hepatic dysfunction. Mol. Cell 6, 87–97 (2000). 10949030

[b8] Czech M. P., Tencerova M., Pedersen D. J. & Aouadi M. Insulin signalling mechanisms for triacylglycerol storage. Diabetologia 56, 949–964 (2013). 23443243 10.1007/s00125-013-2869-1 PMC3652374

[b9] Martinez-Botas J. *et al.* Absence of perilipin results in leanness and reverses obesity in Lepr(db/db) mice. Nat. Genet. 26, 474–479 (2000). 11101849 10.1038/82630

[b10] Zimmermann R. *et al.* Fat mobilization in adipose tissue is promoted by adipose triglyceride lipase. Science 306, 1383–1386 (2004). 15550674 10.1126/science.1100747

[b11] Peirce V., Carobbio S. & Vidal-Puig A. The different shades of fat. Nature 510, 76–83 (2014). 24899307 10.1038/nature13477

[b12] Li T. *et al.* Glucose and insulin induction of bile acid synthesis: mechanisms and implication in diabetes and obesity. J. Biol. Chem. 287, 1861–1873 (2012). 22144677 10.1074/jbc.M111.305789 PMC3265867

[b13] Miki T. *et al.* Defective insulin secretion and enhanced insulin action in KATP channel-deficient mice. Proc. Natl. Acad. Sci. USA 95, 10402–10406 (1998). 9724715 10.1073/pnas.95.18.10402 PMC27906

[b14] Cipriani S., Mencarelli A., Palladino G. & Fiorucci S. FXR activation reverses insulin resistance and lipid abnormalities and protects against liver steatosis in Zucker (fa/fa) obese rats. J. Lipid Res. 51, 771–784 (2010). 19783811 10.1194/jlr.M001602 PMC2842143

[b15] Thomas C. *et al.* TGR5-mediated bile acid sensing controls glucose homeostasis. Cell Metab. 10, 167–177 (2009). 19723493 10.1016/j.cmet.2009.08.001 PMC2739652

[b16] Watanabe M. *et al.* Bile acids induce energy expenditure by promoting intracellular thyroid hormone activation. Nature 439, 484–489 (2006). 16400329 10.1038/nature04330

[b17] Yoon K. H. *et al.* Epidemic obesity and type 2 diabetes in Asia. The Lancet 368, 1681–1688 (2006). 10.1016/S0140-6736(06)69703-1 17098087

[b18] Waki K. *et al.* Alcohol consumption and other risk factors for self-reported diabetes among middle-aged Japanese: a population-based prospective study in the JPHC study cohort I. Diabet. Med. 22, 323–331 (2005). 15717882 10.1111/j.1464-5491.2004.01403.x

[b19] Wong R. H. & Sul H. S. Insulin signaling in fatty acid and fat synthesis: a transcriptional perspective. Curr. Opin. Pharmacol. 10, 684–691 (2010). 20817607 10.1016/j.coph.2010.08.004 PMC3092640

[b20] James D. E., Burleigh K. M. & Kraegen E. W. Time dependence of insulin action in muscle and adipose tissue in the rat *in vivo*. An increasing response in adipose tissue with time. Diabetes 34, 1049–1054 (1985). 3899807 10.2337/diab.34.10.1049

[b21] Yore M. M. *et al.* Discovery of a class of endogenous mammalian lipids with anti-diabetic and anti-inflammatory effects. Cell 159, 318–332 (2014). 25303528 10.1016/j.cell.2014.09.035 PMC4260972

[b22] Kahn S. E., Cooper M. E. & Del Prato S. Pathophysiology and treatment of type 2 diabetes: perspectives on the past, present, and future. The Lancet 383, 1068–1083 (2014). 10.1016/S0140-6736(13)62154-6 PMC4226760 24315620

[b23] Biddinger S. B. *et al.* Hepatic insulin resistance directly promotes formation of cholesterol gallstones. Nat. Med. 14, 778–782 (2008). 18587407 10.1038/nm1785 PMC2753607

[b24] Li T. *et al.* Transgenic expression of cholesterol 7alpha-hydroxylase in the liver prevents high-fat diet-induced obesity and insulin resistance in mice. Hepatology 52, 678–690 (2010). 20623580 10.1002/hep.23721 PMC3700412

[b25] Cao R. *et al.* Bile acids regulate hepatic gluconeogenic genes and farnesoid X receptor via G(alpha)i-protein-coupled receptors and the AKT pathway. J. Lipid Res. 51, 2234–2244 (2010). 20305288 10.1194/jlr.M004929 PMC2903791

[b26] Prawitt J., Caron S. & Staels B. Glucose-lowering effects of intestinal bile acid sequestration through enhancement of splanchnic glucose utilization. Trends Endocrinol. Metab. 25, 235–244 (2014). 24731596 10.1016/j.tem.2014.03.007

[b27] Baba T. *et al.* Estrogen, insulin, and dietary signals cooperatively regulate longevity signals to enhance resistance to oxidative stress in mice. J. Biol. Chem. 280, 16417–16426 (2005). 15713666 10.1074/jbc.M500924200

[b28] Ishikawa K. *et al.* Subcutaneous fat modulates insulin sensitivity in mice by regulating TNF-alpha expression in visceral fat. Horm. Metab. Res. 38, 631–638 (2006). 17075771 10.1055/s-2006-954580

[b29] Rodbell M. Metabolism of isolated fat cells. I. Effects of hormones on glucose metabolism and lipolysis. J. Biol. Chem. 239, 375–380 (1964). 14169133

[b30] Hagio M., Matsumoto M., Fukushima M., Hara H. & Ishizuka S. Improved analysis of bile acids in tissues and intestinal contents of rats using LC/ESI-MS. J. Lipid Res. 50, 173–180 (2009). 18772484 10.1194/jlr.D800041-JLR200

[b31] Muto A. *et al.* Detection of Delta4-3-oxo-steroid 5beta-reductase deficiency by LC-ESI-MS/MS measurement of urinary bile acids. J. Chromatogr. B. Analyt. Technol. Biomed. Life Sci. 900, 24–31 (2012). 10.1016/j.jchromb.2012.05.023 22695323

