# Supplementary material for: Unsuppressed lipolysis in adipocytes is linked with enhanced gluconeogenesis and altered bile acid physiology in InsrP1195L/+ mice fed high-fat-diet
Source: Sci Rep. 2015 Nov 30;5:17565. doi: 10.1038/srep17565 (PMC4663474; doi:10.1038/srep17565)
Supplement: Supplementary Information [file srep17565-s1.pdf]

Unsuppressed lipolysis in adipocytes is linked with enhanced gluconeogenesis and altered bile acid physiology in *Insr*$^{P1195L/+}$ mice fed high-fat-diet

Eun Young Lee,[1¶] Kenichi Sakurai,[2¶] Xilin Zhang,[1] Chitoku Toda,[3] Tomoaki Tanaka,[2] Meizi Jiang,[4] Takuji Shirasawa,[5] Kaori Tachibana,[2] Koutaro Yokote,[2] Antonio Vidal-Puig,[6] Yasuhiko Minokoshi,[3] Takashi Miki[1*]

1 Department of Medical Physiology, 2 Department of Clinical Cell Biology and Medicine, 4 Department of Genome Research and Clinical Application, Chiba University, Graduate School of Medicine, Chiba 260-8670 Japan

3 Department of Developmental Physiology, National Institute for Physiological Sciences, Okazaki 444-8585, Japan

5 Department of Ageing Control, Juntendo University, Graduate School of Medicine. Bunkyo 113-0033, Japan

6 Department of Clinical Biochemistry, Metabolic Research Laboratories, Addenbrooke's Treatment Centre, Institute of Metabolic Science, University of Cambridge, Cambridge CB2 0QQ, UK

¶Equal contributors

*Correspondence: tmiki@faculty.chiba-u.jp

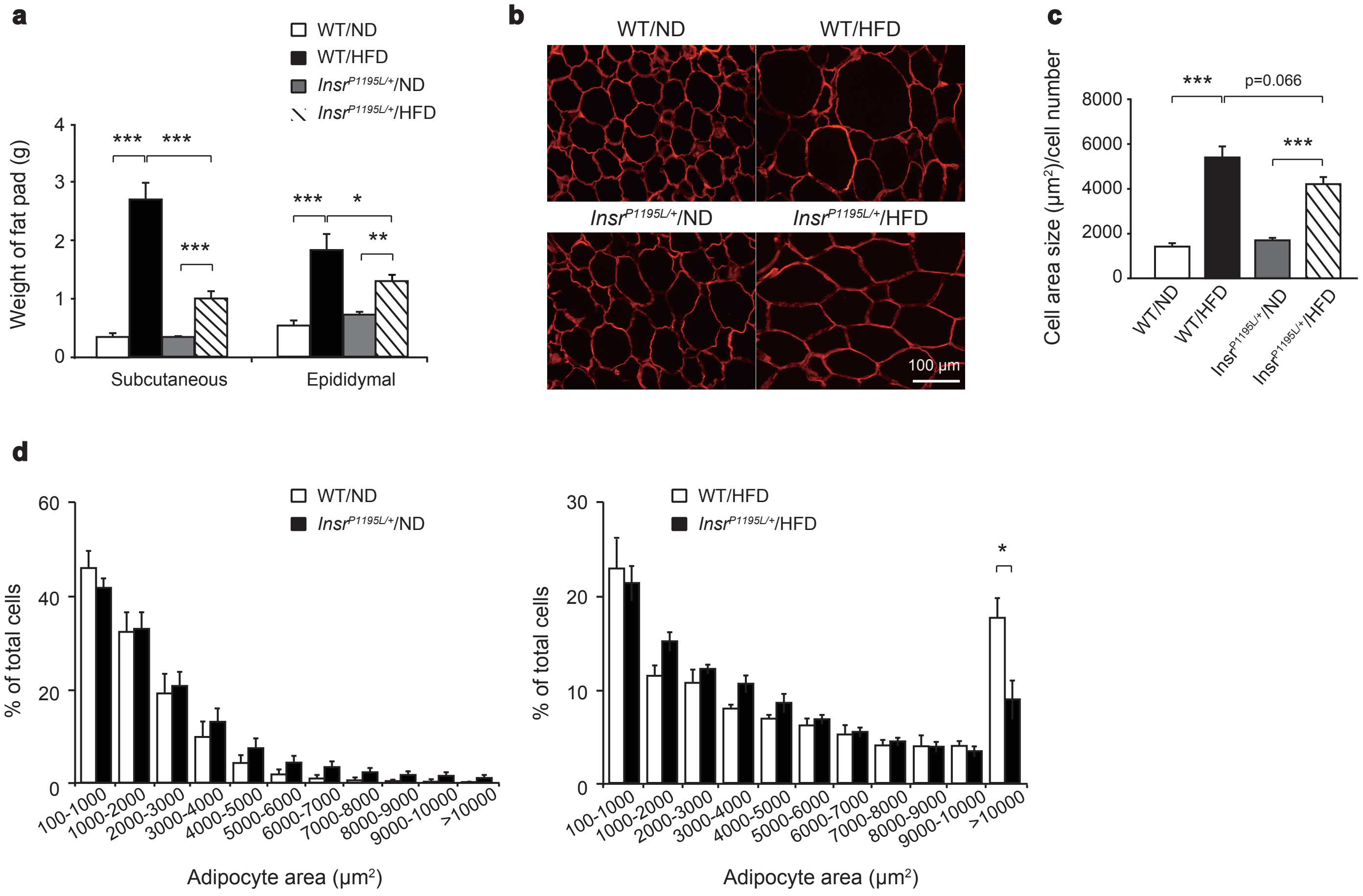

Supplementary Figure S1

Fat weight and adipocyte size. a. Fat weight of the mice at 25 weeks of age (*n* = 3-8). Epididyma fat weight in *Insr*$^{P1195L/+}$/HFD mice was significantly decreased compared with that of WT/HFD mice. b. Immunostaining of epididymal fat tissue with Caveolin-1 (1:1000, BD biosciences). c. Mean adipocyte size. d. Histogram of the cell area of WT/ND and *Insr*$^{P1195L/+}$/ND mice (left) and that of WT/HFD and *Insr*$^{P1195L/+}$/HFD mice (right). The adipocyte size was not different between of WT/ND and *Insr*$^{P1195L/+}$/ND mice (left). By contrast, the number of large (>10000 µm$^{2)}$ adipocytes of *Insr*$^{P1195L/+}$/HFD mice was significantly decreased compared with that of WT/HFD mice (right). Data are mean ± SEM. One-way ANOVA plus Bonferroni *post-hoc* analysis. **P*<0.05, ** *P*<0.01, *** *P*<0.001.

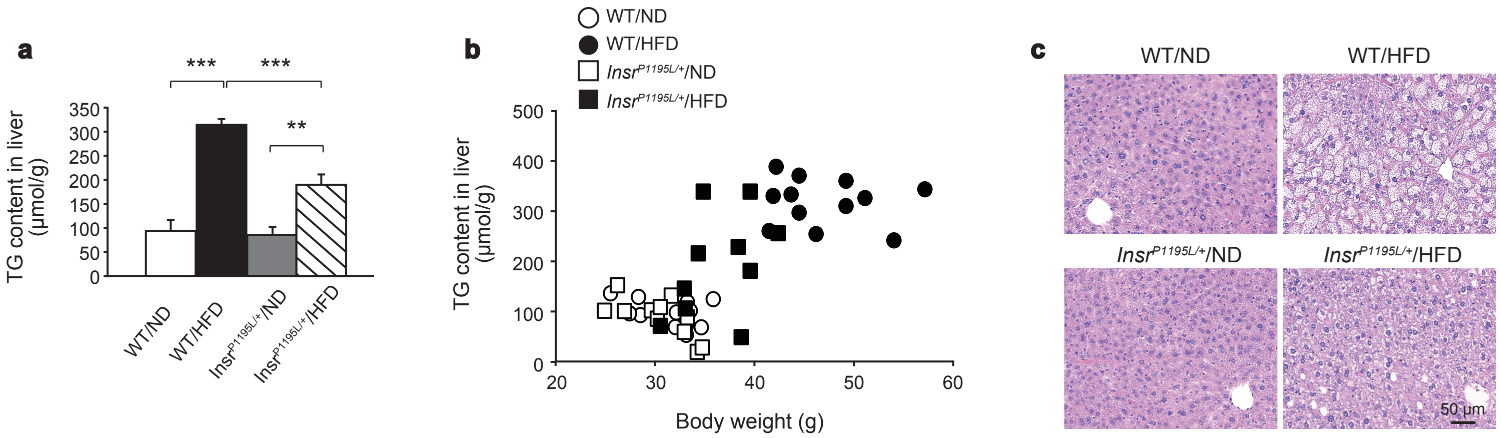

Supplementary Figure S2

Fat accumulation in the liver is reduced in *Insr*$^{P1195L/+}$/HFD mice. (a) TG content in *Insr*$^{P1195L/+}$/HFD liver was significantly less than that in WT/HFD liver (*n* = 10-12 per each group). (b) Relationship between body weight and TG content in liver. (c) HE staining of *Insr*$^{P1195L/+}$/HFD liver shows the amount of lipid droplet is decreased compared with that of WT/HFD liver. Data are mean ± SEM. Two-way ANOVA plus Bonferroni *post-hoc* analysis. ** *P*<0.01, *** *P*<0.001.

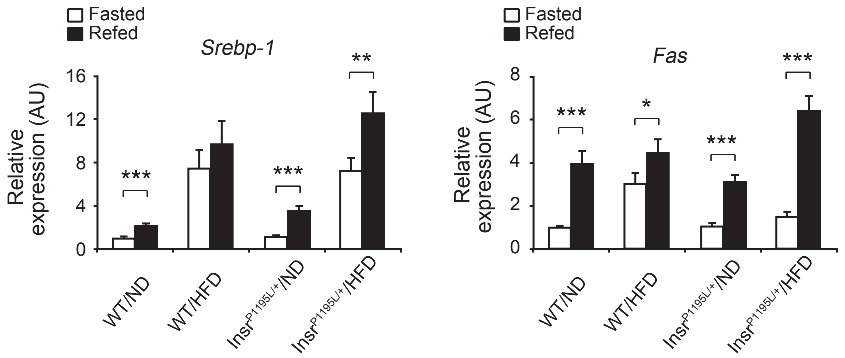

Supplementary Figure S3

mRNA expressions of genes regulated by insulin are not decreased in liver of *Insr*$^{P1195L/+}$/HFD mice. mRNA expressions of *Srebp-1* in *Insr*$^{P1195L/+}$/ND liver is not different from that in WT/ND liver, including its feeding-induced increase. In addition, mRNA expression of *Fas*, a target of *Srebp-1*, is not decreased in *Insr*$^{P1195L/+}$/HFD liver, indicating that induction of *Srebp-1* and *Fas* by refeeding or HFD remains 'sensitive' under insulin resistance in *Insr*$^{P1195L/+}$ mice (n = 10-12 per each group). Data are mean ± SEM. Significance was analyzed by two-tailed Student' s *t*-test between fasted and refed conditions. *P<0.05, ** P<0.01, *** P<0.001.

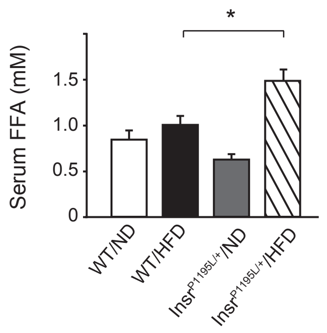

Supplementary Figure S4

Serum FFA levels. Serum FFA levels were measured by using the kit from BioVision, Inc. (San Francisco, CA, USA) (*n* = 8-13 per each group). Data are mean ± SEM. One-way ANOVA plus Bonferroni *post-hoc* analysis. **P*<0.05.

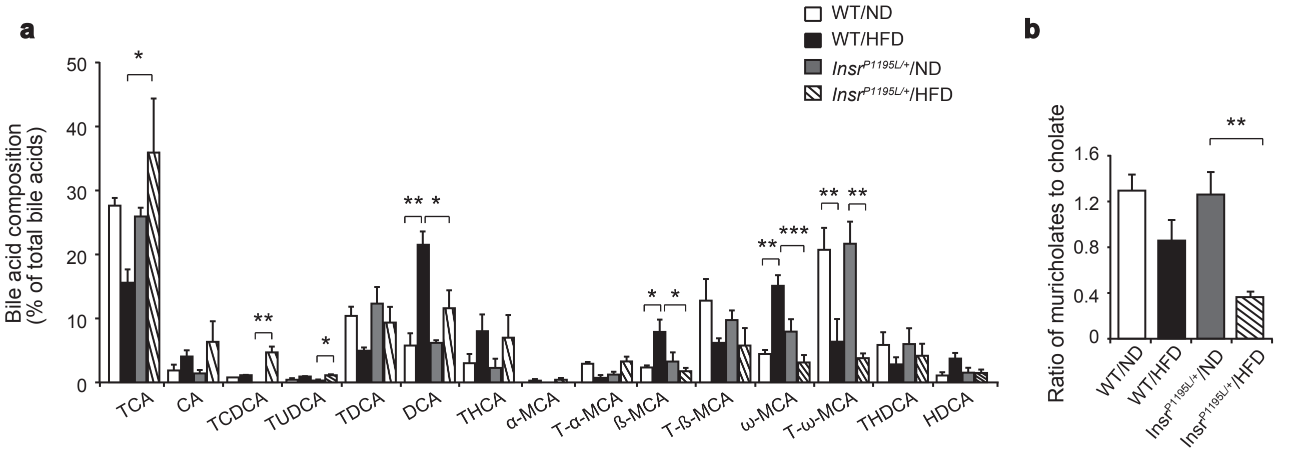

Supplementary Figure S5

Serum bile acid compositon. Measurement of BAs in serum revealed that BA composition was altered in *Insr*$^{P1195L/+}$/HFD mice (n=4 per each group). Data are mean ± SEM. One-way ANOVA plus Bonferroni *post-hoc* analysis. *P<0.05, ** P<0.01, *** P<0.001.

Figure 2h

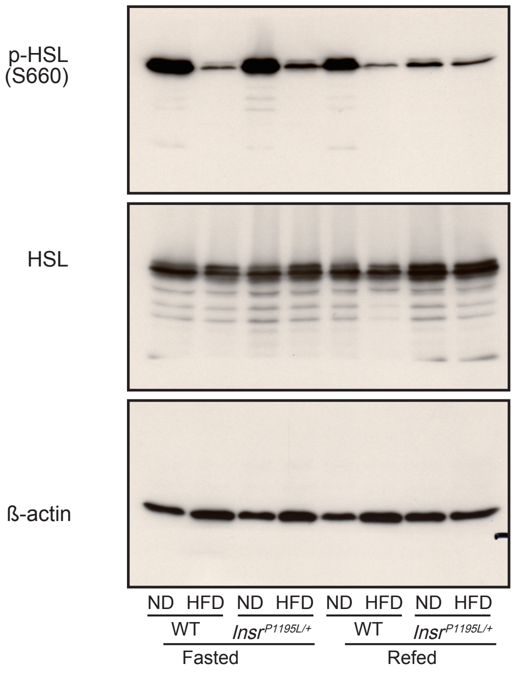

Figure 2i

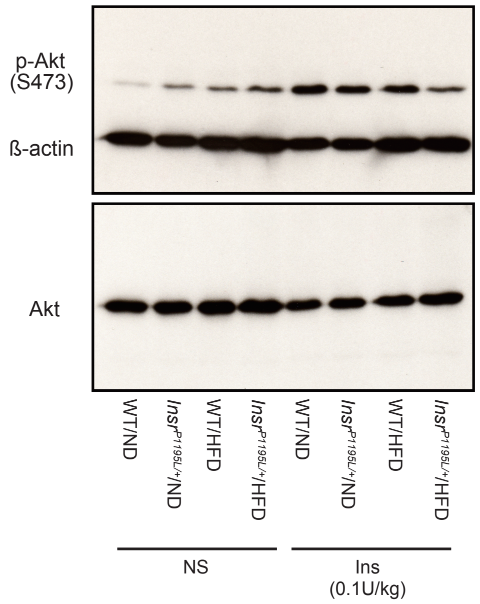

Figure 2j

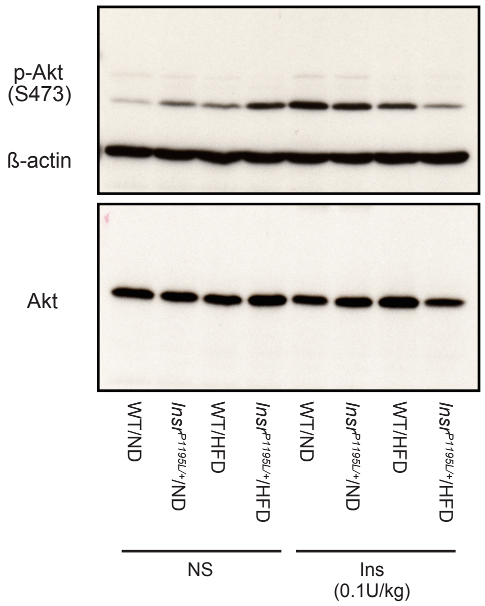

Figure 5d

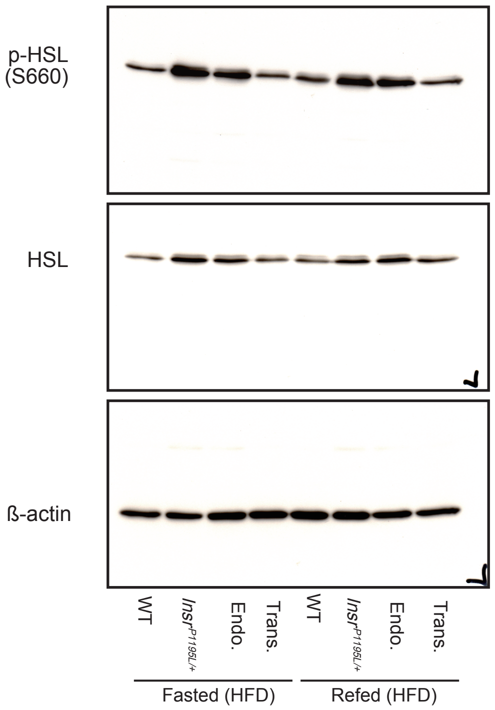

Full-length blots of Figure 2h, 2i,2j,5d.

Supplementary Figure S6
